# Effect of internet-based counselling with a cognitive-behavioural approach on premenstrual syndrome

**DOI:** 10.1186/s13104-022-06222-w

**Published:** 2022-10-12

**Authors:** Sanam Borji-Navan, Sakineh Mohammad-Alizadeh-Charandabi, Khalil Esmaeilpour, Mojgan Mirghafourvand

**Affiliations:** 1 Student Research Committee, Tabriz University of Medical Sciences, Tabriz, Iran; 2 Social Determinants of Health Research Centre, Faculty of Nursing and Midwifery, Tabriz University of Medical Sciences, Tabriz, Iran; 3 Department of Psychology, Faculty of Education and Psychology, University of Tabriz, Tabriz, Iran; 4 Department of Midwifery, Faculty of Nursing and Midwifery, Tabriz University of Medical Sciences, South Shariati Street, 5137975846 Tabriz, Iran

**Keywords:** Cognitive behavior therapy, Internet-based counselling, Premenstrual syndrome, Quality of life

## Abstract

**Objectives:**

To assess the effect of internet-based counselling with a cognitive-behavioural approach on symptom severity of women with premenstrual syndrome (PMS) and their quality of life during the perimenstrual and late follicular phases of the menstrual cycle. Moreover, the PMS-related disability and attitude toward menstruation were investigated as secondary outcomes.

**Data description:**

We provide data generated in a randomized controlled trial with two-parallel arms carried out on 92 female university students aged 18–35 years who had moderate to severe PMS, residing at halls of the Tabriz University of Medical Sciences. PMS severity was assessed during two menstrual cycles before intervention (baseline) and during two cycles just after ending the two-month intervention using Daily Record of Severity of Problems (DRSP) and the quality of life using the Quality of Life Enjoyment and Satisfaction Questionnaire—Short Form (Q-LES-Q-SF) on days 1–2 and 11–13 of the menstrual cycle at the baseline and post-intervention. Also, the PMS-related disability was assessed using Sheehan Disability Scale (SDS) and attitude toward menstruation using Menstrual Attitude Questionnaire (MAQ) at the baseline and post-intervention. Participant satisfaction and views on intervention effectiveness were also assessed using a single Likert question.

## Objective

About 20% of women of reproductive age suffer from premenstrual syndrome (PMS) [1]. The syndrome leads to various consequences, including physical, psychological, behavioral, and social complications [2]. Most previous studies show a positive effect of in-person cognitive-behavioral therapy on PMS severity [3–5]. However, due to some limitations such as distance and time limits, as well as cost constraints, some women are unable to access such therapy [6]. We found only a trial, conducted in a developed country (Germany) [7], with promising results on the positive effect of an alternative method of the therapy named internet-based cognitive-behavioral therapy (ICBT) on PMS. This method has no limitations of the in-person method. Therefore, we aimed to investigate the effect of internet-based counselling with a cognitive-behavioural approach on the PMS severity and the quality of life of Iranian female university students suffering from PMS. In addition, the PMS-related disability and attitude toward menstruation were investigated as secondary outcomes. The details of the experiments and analysis performed and findings based on these data have been published in 2022 [8].

## Data description

The provided data were gathered for a randomized controlled trial with two-parallel arms carried out on 92 eligible university students aged 18–35 years residing at halls of the Tabriz University of Medical Sciences which were equally allocated into the study groups.

The potentially eligible participants, selected using a checklist of initial eligibility requirements, initially completed a socio-demographic and reproductive questionnaire, as well as a validated Persian version of the 19-item Premenstrual Symptoms Screening Tool (PSST) [9]. Those with a positive result in the screening provided written informed consent and filled in the 21-item Daily Record of Severity of Problems (DRSP) [10] during their next menstrual cycle, also completed the 21-item validated Persian version of the Beck Depression Inventory (BDI) [11] on one of the days of their mid-follicular phase (days + 7 to + 10 of the menstrual cycle). After exclusion of those with severe depression, the remaining participants completed the DRSP for the second menstrual cycle, the 3-item Sheehan Disability Scale (SDS) [12] and the 33-item Menstrual Attitude Questionnaire (MAQ) [13] on day 1 or 2 of the second cycle, also 14-item of the Quality-of-Life Enjoyment and Satisfaction Questionnaire—Short Form (Q-LES-Q-SF) [14] twice (on days 1–2 and days 11–13 of the menstrual cycle). Finally, female students with moderate to severe PMS diagnosed by the DRSP were randomized into the groups.

The internet-based educational content for eight sessions was provided during two months for the intervention in a scheduled manner. The control group did not receive any intervention during the study period, but received the content after doing all post-test assessments.

Primary outcomes were the mean score of total PMS symptoms, and quality of life during the perimenstrual and the late follicular phases. Secondary outcomes were the mean scores of sub-scales of DRSP, PMS-related disability, and MAQ domains. All were assessed at baseline post-intervention.

Also, participant satisfaction with the intervention was assessed using a single 5-point Likert question, and their opinions on the effectiveness of intervention using another single 6-point Likert question.

Data have been entered in an SPSS Spreadsheet and analyzed using the software (Table 1).

**Table 1:** Overview of data files/data sets

Label	Name of data file/data set	File types (file extension)	Data repository and identifier (DOI or accession number)
Data file 1	Excel Raw Data-ICBT for PMS	Excel (.xlsx)	Figshare [15] (10.6084/m9.figshare.19082273)
Data file 2	Excel Data-ICBT for PMS	Excel (.xlsx)	Figshare [16] (10.6084/m9.figshare.17711141)
Data set 1	Data analysis Syntax-ICBT for PMS	Word (.docx)	Figshare [17] (10.6084/m9.figshare.19082390)

## Limitations

- It was impossible to blind the participants, healthcare providers, and outcome assessors, who were also the participants.
- We could not recruit any participants from the private residence halls.
- The results may not be generalizable to all women suffering from PMS since the study was included only medical students.
- The long-term effectiveness of the intervention is unknown because we only follow the participants for two menstrual cycles after the end of the intervention.

## Data Availability

The data described in this Data note can be freely and openly accessed on [Figshare] under [10.6084/m9.figshare.19082273, 10.6084/m9.figshare.17711141, 10.6084/m9.figshare.19082390]. Please see Table 1 and references [15–17] for details and links to the data.

## Abbreviations

PMS: Premenstrual syndrome
ICBT: Internet-based cognitive-behavioral therapy
DRSP: Daily Record of Severity of Problems
Q-LES-Q-SF: Quality of Life Enjoyment and Satisfaction Questionnaire—Short Form
PMDD: Premenstrual dysphoric disorder
PSST: Premenstrual Symptoms Screening Tool
SDS: Sheehan Disability Scale
MAQ: Menstrual Attitude Questionnaire

## References

[1] Potter J, Bouyer J, Trussell J, Moreau C (2009). Premenstrual syndrome prevalence and fluctuation over time: results from a French population-based survey. J Womens Health (Larchmt).

[2] Siahbazi S, Montazeri A, Taghizadeh Z, Masoomie R (2018). The consequences of premenstrual syndrome on the quality of life from the perspective of affected women: a qualitative study. J Res Med Dental Sci.

[3] Kancheva Landolt N, Ivanov K (2021). Short report: cognitive behavioral therapy—a primary mode for premenstrual syndrome management: systematic literature review. Psychol Health Med.

[4] Shoaee F, Pouredalati M, Dadshahi S, Parvin P, Bolourian M, Kiani A (2020). Evaluation of non-pharmacological strategies, therapeutic and cognitive-behavioral interventions in the treatment of premenstrual syndrome: a review study. International Journal of Pediatrics..

[5] Başoğul C, Aydın Özkan S, Karaca T (2020). The effects of psychoeducation based on the cognitive-behavioral approach on premenstrual syndrome symptoms: a randomized controlled trial. Perspect Psychiatr Care.

[6] Mohr DC, Ho J, Duffecy J, Reifler D, Sokol L, Burns MN (2012). Effect of telephone-administered vs face-to-face cognitive behavioral therapy on adherence to therapy and depression outcomes among primary care patients: a randomized trial. JAMA.

[7] Weise C, Kaiser G, Janda C, Kues JN, Andersson G, Strahler J (2019). Internet-based cognitive-behavioural intervention for women with premenstrual dysphoric disorder: a randomized controlled trial. Psychother Psychosom.

[8] Borji-Navan S, Mohammad-Alizadeh-Charandabi S, Esmaeilpour K, Mirghafourvand M, Ahmadian-Khooinarood A (2022). Internet-based cognitive-behavioral therapy for premenstrual syndrome: a randomized controlled trial. BMC Womens Health.

[9] Hariri FZ, Moghaddam-Banaem L, Siah Bazi S, Saki Malehi A, Montazeri A (2013). The Iranian version of the Premenstrual Symptoms Screening Tool (PSST): a validation study. Arch Womens Ment Health.

[10] Endicott J, Nee J, Harrison W (2006). Daily Record of Severity of Problems (DRSP): reliability and validity. Arch Women Ment Health.

[11] Ghassemzadeh H, Mojtabai R, Karamghadiri N, Ebrahimkhani N (2005). Psychometric properties of a Persian-language version of the Beck Depression Inventory—second edition: BDI-II-PERSIAN. Depress Anxiety.

[12] Sheehan DV, Harnett-Sheehan K, Raj BA (1996). The measurement of disability. Int Clin Psychopharmacol.

[13] Brooks-Gunn J, Ruble DN (1980). The menstrual attitude questionnaire. Psychosom Med.

[14] Quick F, Mohammad-Alizadeh-Charandabi S, Mirghafourvand M (2019). Primary dysmenorrhea with and without premenstrual syndrome: variation in quality of life over menstrual phases. Qual Life Res.

[15] Borji-Navan S, Mohammad-Alizadeh-Charandabi S, Esmaeilpour K, Mirghafourvand M (2022).

[16] Borji-Navan S, Mohammad-Alizadeh-Charandabi S, Esmaeilpour K, Mirghafourvand M (2022).

[17] Borji-Navan S, Mohammad-Alizadeh-Charandabi S, Esmaeilpour K, Mirghafourvand M (2022).

